# Available studies fail to provide strong evidence of increased risk of diarrhea mortality due to measles in the period 4–26 weeks after measles rash onset

**DOI:** 10.1186/s12889-017-4745-2

**Published:** 2017-11-07

**Authors:** Bianca D. Jackson, Robert E. Black

**Affiliations:** Department of International Health, Bloomberg School of Public Health, Johns Hopkins University, Baltimore, MD 21205 USA

**Keywords:** Measles, Diarrhea, Mortality, Delayed measles mortality, Post-measles diarrhea

## Abstract

**Background:**

Measles vaccination effectiveness studies showed dramatic decreases in all-cause mortality in excess of what would be expected from the prevention of measles disease alone. This invited speculation that measles infection may increase the risk of diarrhea morbidity and mortality subsequent to the acute phase of the disease. The aim of the present systematic review is to summarize the existing evidence in the publically available literature pertaining to the putative causal link between measles and diarrhea in the period 4–26 weeks following measles rash onset.

**Methods:**

We searched the PubMed, Embase, Open Grey and Grey Literature Report databases for relevant literature using broad search terms. Prospective, retrospective and case-control studies in low- and middle-income countries involving children under five wherein relevant evidence were presented were included. Data were extracted from the articles and summarized.

**Results:**

Fifty abstracts retrieved through the database searches met the initial screening criteria. Twelve additional documents were identified by review of the references of the documents found in the initial searches. Six documents representing five unique studies that presented evidence relevant to the research question were found. Four of the included studies took place in Bangladesh. One of the included studies took place in Sudan. Some measles vaccine effectiveness studies show lower diarrhea morbidity and mortality among the vaccinated. However, children who received vaccine may have differed in important ways from children who did not, such as health service utilization. Additionally, cohort studies following unvaccinated children showed no difference in diarrhea morbidity and mortality between cases and controls more than 4 weeks after measles rash onset. One study showed some evidence that severe measles may predispose children to gastroenteritis, but was not able to show a corresponding increase in the risk of diarrhea mortality.

**Conclusions:**

The available evidence suggests that the risk of measles-associated diarrhea mortality is largely limited to the 5-week period 1 week prior to and 4 weeks after measles rash onset, and that there is no increased risk of diarrhea mortality in the longer-term caused by measles.

## Background

It is well-documented that diarrhea is a common complication of measles infection in the acute phase of the disease, the 5-week period starting 1 week before rash onset and ending 4 weeks after rash onset [1–12]. However, it is unclear if the increased risk of diarrhea extends longer than 1 month after rash onset. Shahid et al., found in a study in Bangladesh, that children were at increased risk of dysentery for 5 months following measles infection [13]. This led Feachem et al. to speculate that there may be a “post-measles diarrhea” phenomenon that occurs in the 4–26 weeks following measles rash onset [14]. It would appear to support their hypothesis that measles vaccine trials have shown decreases in all-cause mortality in vaccinated children compared to unvaccinated children, which exceed the expected reduction in mortality from prevention of measles alone [15]. In these studies, measles vaccination seemed to have an effect on mortality from other causes, particularly diarrhea [16].

If there is a demonstrable effect of measles on diarrhea risk after the acute phase of the disease, it would be relevant to the efforts to model the impact of measles vaccination on large populations. The Lives Saved Tool (LiST) is a software program that is used to model the impact of maternal and child health interventions, such as childhood vaccinations [17]. Currently in the LiST model, increasing coverage of measles vaccination results in decreased measles mortality, but not decreased diarrhea mortality.

Diarrhea deaths that occur during the acute phase of measles are typically attributed to measles rather than diarrhea, according to ICSD rules [18]. Because of this rule regarding the classification of causes of death, diarrhea deaths due to measles up to 4 weeks after measles rash onset are already captured as measles deaths. The ambiguity, therefore, exists primarily regarding the diarrhea deaths that occur after this period and whether a proportion of those deaths should be attributed to measles. Thus, this review was undertaken to assess the existing evidence of increased risk of delayed diarrhea mortality that can be attributed to measles, for the purpose of ensuring that the full impact of measles vaccination is captured by the LiST model. This review defines the “post-measles” period as the period 4–26 weeks post measles rash onset, however, it should be noted that the definition varies in the literature.

## Methods

### Literature search

The PubMed, Embase, Open Grey and Grey Literature Report databases were searched using the search terms specified in Table 1. The search terms were designed to be more broad than precise. Relevance to the research question was determined by review of the abstracts. The full texts of articles with relevant abstracts were reviewed to determine if the articles should be included, according to the inclusion and exclusion criteria.

**Table 1:** PubMed search terms and number of results

Database	Search Terms	Number of results
PubMed	((“Measles”[Mesh] OR “measles”[tiab]) AND (“Diarrhea”[Mesh] OR “diarrhea”[tiab] OR “diarrheas”[tiab] OR “flux”[tiab] OR “loose stool”[tiab] OR “loose stools”[tiab])) AND (“Morbidity”[Mesh] OR “morbidity”[tiab] OR “morbidities”[tiab] OR “Mortality”[Mesh] OR “mortality”[tiab] OR “mortalities”[tiab] OR “death rate”[tiab] OR “fatality rate”[tiab] OR “vaccine”[tiab])	341
Embase	‘measles’/exp. AND (‘diarrhea’/exp. OR (loose:ab,ti AND stool:ab,ti) OR (loose:ab,ti AND stools:ab,ti)) AND (morbidity:ab,ti OR mortality:ab,ti OR mortalities:ab,ti OR (mortality:ab,ti AND rate:ab,ti) OR (death:ab,ti AND rate:ab,ti) OR (fatality:ab,ti AND rate:ab,ti))	271
Open Grey	“measles”	114
Grey Literature Report	“measles”	6

### Inclusion and exclusion criteria

The review included randomized controlled trials (RCTs), longitudinal observational studies, and case-control studies in children under 5 years of age where the exposure was measles infection or measles vaccination status and the outcome was diarrhea morbidity, diarrhea mortality, or all-cause mortality in the period 4–26 weeks after measles rash onset. Studies involving only persons over 5 years of age were excluded. Studies that included both children under 5 years of age and over 5 years of age were included. Studies were not excluded on the basis of publication date or the language in which they were written.

### Data extraction

One reviewer extracted the following data from relevant studies: year, country, population, design, number of subjects, age range, intervention, duration and timing, length of follow-up, recall period, exposure, outcome, statistical model, covariates, results, effect estimate, limitations, and quality of the evidence.

## Results

### Literature search

A total of 732 documents were identified in the database search. Seventy-one abstracts met the initial screening criteria, and those documents were retrieved for full text review. Twelve additional documents were identified by review of the references of the documents retrieved in the initial search and retrieved for full-text review. Six articles representing five unique studies were deemed relevant based on the inclusion and exclusion criteria (Table 2). Searches in two grey literature databases returned no relevant documents.

**Table 2:** Summary of articles included in this review

First Author	Year	Country	Population	Design	*n*	Ages	Intervention	Statistical Model	Covariates
Aaby^1^	2003	Bangladesh	Rural	Re-analysis of prospective study	16,268	9 months - 60 months	Measles vaccination	Cox proportional hazards	Matched for age. Adjusted for sex, number of siblings, maternal education, size of dwelling
Akramuzzaman	2000	Bangladesh	Urban	Prospective	254	6 months–143 months	None	Relative risk	Age, sex, stunting, vaccination status, mother’s education, number of children in the household,electricity supply, and source of drinking water, SES
Ibrahim	2002	Sudan	Suburban (displaced persons)	Prospective	187	4–168 months	None	Chi-square	None
Koenig	1990	Bangladesh	Rural	Prospective	16,270	9 months - 60 months	Measles vaccination	Cox proportional hazards	Birth order, sex, maternal education level, area of household (SES)
Koster	1981	Bangladesh	Rural	Prospective	5775	<4 years	None	Histogram	N/A
Shahid	1983	Bangladesh	Rural	Retrospective	77	<2 years	None	Crude rates	N/A

### Measles vaccine effectiveness study

In a study by Koenig et al. in Bangladesh from 1982 to 1985, 8134 children were immunized against measles and matched with unimmunized controls in a comparison area. They found that mortality rates for immunized children were 46% lower than for unimmunized children [1]. This is far in excess of the mortality reduction that would be expected from prevention of measles mortality alone. Reanalysis of Koenig et al.’s data by Aaby et al. in 2003 revealed that the vaccine effectiveness against death, adjusting for sex, number of siblings, maternal education and size of dwelling, only decreased from 49% to 43% after excluding deaths attributed to measles. They found that measles immunization also had a protective effect against diarrhea and dysentery, mortality ratio (MR) = 0.54 95% CI (0.42, 0.71), and other causes of death in children aged 9–60 months. However, prevention of post-measles mortality (>45 days post-measles) is unlikely to be the explanation for the unexpectedly large protective effect seen in this study. While the hazard ratio for measles deaths among the unvaccinated children is 17.35, 95% CI (11.9, 25.3), in the 0–45 days post-measles period, the hazard ratio becomes non-significant: 2.35, 95% CI (0.95, 5.84). Furthermore, in the 93–365 days post-measles period, the measles cases have lower hazard of death 0.40 95% CI (0.16, 0.98), and after 1 year post-measles, there is no significant difference in survival between unimmunized measles cases and unimmunized paired controls. Thus, measles immunization may prevent measles-associated diarrhea that occurs within 45 days of measles disease, but this study showed no significant evidence that measles immunization prevents mortality that occurs later than 45 days after measles disease. Additionally, children in this study were not randomized to vaccination/non-vaccination and the groups may differ in important ways, such as health system usage. Koenig et al. reported that there was no significant difference between the groups in terms of “use of contraception” by the mothers and presented this as evidence that selectivity in health behavior is unlikely to have affected the observed results. However, no indicators of care-seeking or usage of child health interventions were reported and it is unclear if use of contraception alone can be indicative of health care system usage for children.

### Cohort study of populations unimmunized against measles

**Fig. 1:**
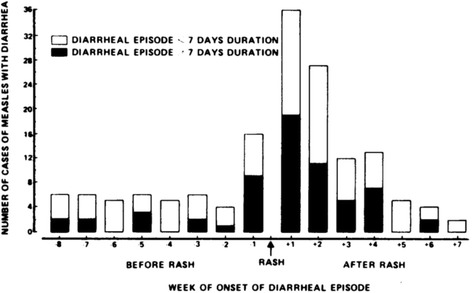
The onset and duration of 149 episodes of diarrhea in relation to the onset of measles rash in 119 measles cases from Koster et al.’s 1981 study “Synergistic impact of measles and diarrhea on nutrition and mortality in Bangladesh” [2]. Diarrheal episodes less than 7 days long are shaded in white. Diarrheal episodes longer than 7 days are shaded in black. Permission to reproduce figure granted by Oxford University Press 06/12/2016 License Number 4003101368875

Koster et al. conducted prospective household surveillance of 5775 children in rural Bangladesh for 12 months (August 1975–July 1976), monitoring diarrhea and measles mortality and morbidity [2]. They found that among measles cases, there was a clear, marked increase in episodes of diarrhea in the 5-week period starting 1 week before measles rash onset and ending 4 weeks after measles rash onset. After 4 weeks, diarrheal episode incidence appeared to return to pre-measles levels (Fig. 1). Thus, these authors found that the increased risk of diarrhea caused by measles seems to be limited to the period immediately following rash onset. However, the authors did not present results for the period 8–26 weeks following rash onset.

A retrospective case cohort study published in 1983 by Shahid et al. of the long-term complications of measles in children under 2 years of age in rural Bangladesh found higher risk of mucoid diarrhea (*p* < 0.01) and bloody diarrhea (*p* < 0.01) among measles cases during an outbreak that occurred March–April 1980 compared to controls [13]. There was no statistically significant difference in the incidence of watery diarrhea between the groups. The researchers used demographic surveillance records to select the case cohort (*n* = 76) and control cohort (*n* = 77) and interviewed case and control children’s household members July 28th – August 15th 1980. The number of episodes of diarrhea occurring after the disappearance of the rash (cases) or after March 31st 1980 (controls) were recorded. However, the authors did not segregate the number of diarrhea episodes by time after measles rash onset, so it is unclear how many of the diarrhea episodes occurred in the first month post rash onset and how many occurred in the 4–28 weeks post rash onset. If the natural history of disease for these cases resembles that which was seen by Aaby et al. and Koster et al., it may be that the excess diarrhea episodes occurred during the acute phase of the disease, and that there is no significant difference in the number of diarrhea episodes in the months following the acute phase of the disease.

**Fig. 2:**
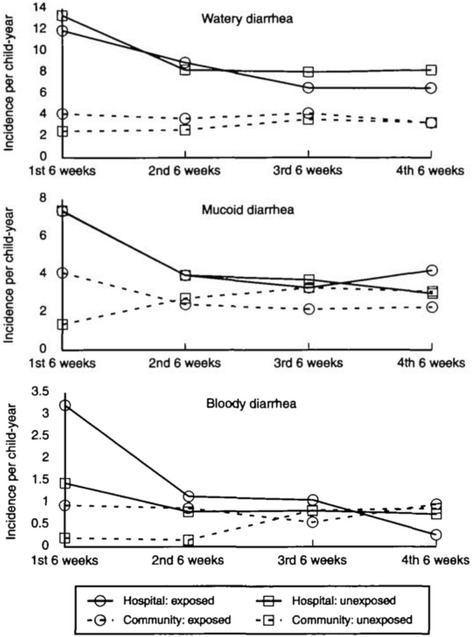
The incidence of three types of diarrhea in Akramuzzaman et al.’s cohort study of measles cases and matched controls [19]. The study included a hospital cohort and community cohort of children aged 6 months – 143 months who were followed up weekly for 24 weeks after recruitment. Diarrhea incidence is calculated at 6-week intervals. Permission to reproduce figure granted by WHO 20/12/2016 Permission Request Number 215071

A later cohort study in Dhaka, Bangladesh by Akramuzzaman et al. in 1995–1996 of 254 children with measles and their age-matched unexposed controls found increased risk of bloody diarrhea in hospitalized measles cases compared to hospitalized controls, aRR = 2.7 (95% CI: 1.4, 5.1) and also in community cases compared to community controls, aRR = 4.1 (95% CI: 1.1, 14.6) [19]. However, the increased risk was limited to the first 6-week period post-measles (Fig. 2). There was no significant difference in watery diarrhea incidence between cases and controls in either the hospital or community cohorts. There was a significant increased incidence of mucoid diarrhea among cases compared to controls in the community cohort (adjusted RR = 2.4, *p* = 0.003), but there was no significant difference between cases and controls in the hospital cohort. The increased risk in the cases in the community cohort also did not persist after the first six-week period of follow-up (Fig. 2). In fact, the authors found no increased risk of diarrhea morbidity of any type that persisted beyond 6 weeks of follow-up during their total 24 weeks of follow-up.

### Cohort study of measles in population of internally displaced persons

A study conducted by Ibrahim et al. in suburban Khartoum, Sudan from March 1997 to July 1999 in a population composed mostly of internally displaced persons, followed a cohort of measles cases and non-measles rash cases for 12 months [20]. The ages of children in the cohort ranged from 4 months to 14 years. The authors classified cases as “severe measles” cases if they developed associated complications that required hospitalization. In order to ensure that they would be able to compare outcomes between severe and uncomplicated measles cases, they recruited severe measles cases from hospitals that were not part of the cohort. They followed the cohort and additional severe cases for 12 months. There was no significant difference in mortality between measles cases and non-measles cases observed at any of the time points during follow-up. At 1 month of follow-up and 6 months of follow-up, there was a significantly higher rate of gastroenteritis among severe cases of measles (7/22, 32%), compared to uncomplicated cases of measles (4/42, 9.5%). It is not stated by the authors what proportion of the gastroenteritis cases experienced diarrhea and how many experienced only vomiting or other gastrointestinal symptoms. There was no significant difference in incidence of gastroenteritis at 12 months of follow-up between the groups, and there was no significant difference in mortality at any time point during follow-up. Only three deaths were observed in the cohort and severe cases during 12 months of follow-up. Two were in the severe measles group by the 1-month follow-up visit and one between the 1-month and 6-month follow up visits. One death occurred in the uncomplicated measles group between the 1-month and 6-month follow-up visits. No deaths occurred in the non-measles rash group. Notably, 24 of the 49 severe measles cases were lost to follow-up before the 1-month visit, compared to 4 among the 52 uncomplicated measles cases and 0 among the 33 non-measles cases.

## Discussion

There is a paucity of studies addressing the question of whether measles increases the risk of diarrhea morbidity and mortality in the period 4–26 weeks after measles rash onset. However, the data that exists suggest that the increased risk of diarrhea mortality is limited to the acute phase of the disease.

The strongest evidence for this conclusion comes from the studies by Koster et al. and Akramuzzaman et al., where they presented diarrhea incidence as a function of time since measles rash [2, 19]. In Koster et al.’s study participants, the number of diarrhea episodes was elevated during the acute phase of measles infection, peaked at 1 week post-rash and returned to pre-infection levels by week 5 post-rash (Fig. 1). Similarly, Akramuzzaman et al. presented diarrhea incidence data for 24 weeks after measles disease and their results are consistent with the conclusion that increased diarrhea risk is limited to the acute phase of measles infection. Ibrahim et al.’s study also showed no difference in the incidence of gastroenteritis between measles cases and non-measles rash cases at 6 months and 12 months of follow-up. They did find a statistically significant increased risk of gastroenteritis among severe measles cases compared to uncomplicated measles cases at 6 months after measles rash onset. However, an unspecified number of severe measles cases were drawn from hospital cases outside of the cohort to which they were compared, so it is unclear if the comparison is valid. Furthermore, almost half of the severe cases were lost to follow-up before by 1 month after the initial visit, so definitive conclusions cannot be drawn from this subset. The results of this study did not show an increased risk of death among measles cases, compared to non-measles rash cases, or severe measles cases compared to uncomplicated measles cases at 1 month, 6 months or 12 months of follow-up. However, the sample size was small and many individuals were lost to follow-up, especially in the severe measles group, which may have prevented the detection of a long-term effect of measles on diarrhea mortality, if such an effect exists.

Thus, the available evidence does not support the hypothesis that the excess decrease in mortality seen in measles vaccination studies such as Koenig et al.’s can be explained by prevention of delayed measles-associated diarrhea mortality. Alternative explanations for the observed decrease could include uncontrolled differences between the vaccinated and unvaccinated groups, in terms of health status, socio-economic status, health system utilization, and other factors. The act of vaccinating the children may also have increased their number of health system interactions relative to their unvaccinated counterparts, providing more opportunities for parents and caregivers to seek advice, care, or referrals from health workers. It is also possible that there is some immune system activation stimulated by the measles vaccine that provides non-specific protection from other diseases. A study in Bangladesh showed increased expression of immune system cytokine, interleukin 2, and lymphocyte proliferation markers (CD 25, CD 69, CD71), as well as higher concentrations of natural killer lymphocytes in vaccinated children 24 weeks following vaccination, but it is unclear whether this immune activation necessarily leads to enhanced non-specific protection from disease [21]. Further research is needed regarding the possibility of non-specific beneficial or deleterious effects of vaccines on infectious disease morbidity and mortality [22]. However, conducting an RCT to address this subject would require depriving children of a proven, efficacious vaccine [23]. Thus, it is unlikely that future studies will be able to satisfactorily show if the measles vaccine has non-specific effects on child mortality from causes unrelated to measles infection.

### Limitations

Five out of the six studies that met the inclusion/exclusion criteria were conducted in Bangladesh, so it is unclear if the conclusions made by this review are generalizable to other settings.

## Conclusions

Measles-associated diarrhea morbidity and mortality is largely limited to the acute phase of measles infection. There is insufficient evidence to support the hypothesis that measles increases the risk of diarrhea morbidity and mortality more than 4 weeks after measles rash onset.

## Abbreviations

GRADE: Grading of recommendations assessment, development and evaluation
LiST: Lives saved tool
RCT: Randomized controlled trial

## References

[1] Koenig M, Khan M, Wojtyniak B, Clemens JD, Chakraborty J, Fauveau V (1990). Impact of measles vaccination on childhood mortality in rural Bangladesh. Bull World Health Organ.

[2] Koster FT, Curlin GC, Aziz KNA, Haque A (1981). Synergistic impact of measles and diarrhoea on nutrition and mortality in Bangladesh. Bull World Health Organ.

[3] Grais RF, Dubray C, Gerstl S, Guthmann JP, Djibo A, Nargaye KD (2007). Unacceptably high mortality related to measles epidemics in Niger, Nigeria, and Chad. PLoS Med.

[4] Clemens JD, Stanton BF, Chakraborty J, Chowdhury S, Rao MR, Ali M (1988). Measles vaccination and childhood mortality in rural Bangladesh. Am J Epidemiol.

[5] Greenberg ABL, Sack RB, Budge E, Gutierrez M, Visberg A, Yi A (1991). Measles-associated Diarrhea in hospitalized children in lima, Peru : pathogenic agents and impact on growth. J Infect Dis.

[6] John TJ, Joseph A, George TI, Radhakrishnan J, Singh RPD, George K (1980). Epidemiology and prevention of measles in rural south India. Indian J Med Res.

[7] Marufu T, Siziya S, Mudambo KST (2008). Factors associated with secular trends in mortality attributed to measles in Gweru, Zimbabwe, in 1967-89. J Trop Pediatr.

[8] Marufu T, Siziya S (1998). Secular changes in rates of respiratory complications and diarrhoea among measles cases. J Trop Pediatr.

[9] Marufu T, Siziya S, Murugasampillay S, Mason E, Manyame B, Tshimanga M (1997). Measles complications: the importance of their management in reducing mortality attributed to measles. Cent Afr J Med.

[10] Narain JP, Khare S, Rana SRS, Banerjee KB (1989). Epidemic measles in an isolated unvaccinated population, India. Int J Epidemiol.

[11] Scrimshaw NS, Salomon JB, Bruch HA, Gordon JE (1966). Studies of diarrheal disease in central America. Am J Trop Med Hyg.

[12] Taufan I, Rampengan T (1991). Measles enteritis in Gunuug Wenang general hospital Manado. Paediatr Indones.

[13] Shahid NS, Clauquin P, Shaikh K, Zimicki S (1983). Long-term complications of measles in rural Bangladesh. J Trop Med Hyg.

[14] Feachem RG, Koblinsky MA (1983). Interventions for the control of diarrhoeal diseases among young children: measles immunization. Bull World Health Organ.

[15] Aaby P, Samb B, Simondon F, Marie A, Seek C, Knudsen K (1995). Non-specific beneficial effect of measles Immunisation : analysis of mortality studies from developing countries. Br Med J.

[16] Aaby P, Bhuiya A, Nahar L, Knudsen K, de Francisco A, Strong M. The survival benefit of measles immunization may not be explained entirely by the prevention of measles disease: a community study from rural Bangladesh. Int J Epidemiol. 2003;32:106–15. 10.1093/ije/dyg005. 10.1093/ije/dyg005 12690020

[17] Walker N, Tam Y, Friberg IK (2013). Overview of the lives saved tool (LiST). BMC Public Health.

[18] WHO. International Statistical Classification of Diseases and Related Health Problems 10th Revision. 2016. http://apps.who.int/classifications/icd10/browse/2016/en#/B05.

[19] Akramuzzaman SM, Cutts FT, Wheeler JG, Hossain MJ (2000). Increased childhood morbidity after measles is short-term in urban Bangladesh. Am J Epidemiol.

[20] Ibrahim SA, Mustafa OM, Mukhtar MM, Saleh EA, El Mubarak HS, Abdallah A (2002). Measles in suburban Khartoum: an epidemiological and clinical study. Trop Med Int Heal.

[21] Schnorr JJ, Cutts FT, Wheeler JG, Akramuzzaman SM, Alam MS, Azim T (2001). Immune modulation after measles vaccination of 6-9 months old Bangladeshi infants. Vaccine.

[22] World Health Organization. Weekly epidemiological record: relevé épidémiologique hebdomadaire. Wkly Epidemiol Rec. 2014;89:221–36. 10.1371/jour.

[23] Demicheli V, Rivetti A, Mg D, DP C, Demicheli V, Rivetti A (2012). Vaccines for measles, mumps and rubella in children ( review ) vaccines for measles, mumps and rubella in children. Cochrane Database Syst Rev.

